# Complex Patterns of Genomic Heterogeneity Identified in 42 Tumor Samples and ctDNA of a Pulmonary Atypical Carcinoid Patient

**DOI:** 10.1158/2767-9764.CRC-22-0101

**Published:** 2023-01-10

**Authors:** Tamsin J. Robb, Peter Tsai, Sandra Fitzgerald, Paula Shields, Pascalene S. Houseman, Rachna Patel, Vicky Fan, Ben Curran, Rexson Tse, Jacklyn Ting, Nicole Kramer, Braden J. Woodhouse, Esther Coats, Polona Le Quesne Stabej, Jane Reeve, Kate Parker, Ben Lawrence, Cherie Blenkiron, Cristin G. Print

**Affiliations:** 1 Department of Molecular Medicine and Pathology, Faculty of Medical and Health Sciences, University of Auckland, Auckland, New Zealand.; 2 Maurice Wilkins Centre for Biodiscovery hosted by the University of Auckland, Auckland, New Zealand.; 3 Department of Forensic Pathology, LabPLUS, Auckland, New Zealand.; 4 Department of Anatomical Pathology, LabPLUS, Auckland, New Zealand.; 5 Surgical Pathology Unit, North Shore Hospital, Waitemata District Health Board, Auckland, New Zealand.; 6 Department of Oncology, Faculty of Medicine and Health Sciences, University of Auckland, Auckland, New Zealand.; 7 Radiology, Auckland District Health Board, Auckland, New Zealand.; 8 Auckland Cancer Society Research Centre, Faculty of Medical and Health Sciences, University of Auckland, Auckland, New Zealand.

## Abstract

Tumor evolution underlies many challenges facing precision oncology, and improving our understanding has the potential to improve clinical care. This study represents a rare opportunity to study tumor heterogeneity and evolution in a patient with an understudied cancer type.

A patient with pulmonary atypical carcinoid, a neuroendocrine tumor, metastatic to 90 sites, requested and consented to donate tissues for research. 42 tumor samples collected at rapid autopsy from 14 anatomically distinct sites were analyzed through DNA whole-exome sequencing and RNA sequencing, and five analyzed through linked-read sequencing. Targeted DNA sequencing was completed on two clinical tissue biopsies and one blood plasma sample.

Chromosomal alterations and gene variants accumulated over time, and specific chromosomal alterations preceded the single predicted gene driver variant (*ARID1A)*. At the time of autopsy, all sites shared the gain of one copy of Chr 5, loss of one copy of Chr 6 and 21, chromothripsis of one copy of Chr 11, and 39 small variants. Two tumor clones (carrying additional variants) were detected at metastatic sites, and occasionally in different regions of the same organ (e.g., within the pancreas). Circulating tumor DNA (ctDNA) sequencing detected shared tumor variants in the blood plasma and captured marked genomic heterogeneity, including all metastatic clones but few private tumor variants.

This study describes genomic tumor evolution and dissemination of a pulmonary atypical carcinoid donated by a single generous patient. It highlights the critical role of chromosomal alterations in tumor initiation and explores the potential of ctDNA analysis to represent genomically heterogeneous disease.

**Significance:**

DNA sequencing data from tumor samples and blood plasma from a single patient highlighted the critical early role of chromosomal alterations in atypical carcinoid tumor development. Common tumor variants were readily detected in the blood plasma, unlike emerging tumor variants, which has implications for using ctDNA to capture cancer evolution.

## Introduction

Tumor evolution underlies many of the most pressing challenges facing clinical precision oncology today, and a better understanding of this process has the potential to improve clinical care. We present a unique example of tumor evolution in an uncommon tumor type not previously featured in such studies, a pulmonary atypical carcinoid [an intermediate grade neuroendocrine tumor (NET)].

NETs arise from hormone-producing cells of the neuroendocrine system located throughout the body, and are highly heterogeneous, both genetically and pathologically. While once considered rare tumors (1) and thought to be indolent in nature (2), we now recognize that NETs have an age-adjusted incidence of 6.2 cases per 100,000 in the country where this study was undertaken, New Zealand (3), similar to the incidence of ovarian and cervical cancers (4). Pulmonary atypical carcinoids are mitotically active well-differentiated NETs, with one in five presenting with distant metastatic disease at diagnosis (5). They feature relatively few DNA variants, with recurrent variants occurring in chromatin remodeling genes such as *MEN1* and *ARID1A* [reported in 25% and 10% of studied atypical carcinoids, respectively (6)]. In general, NETs feature few small DNA variants and often instead have large-scale chromosomal changes; for example, around 25% of pancreatic NETs lose a suite of 10 chromosomes, and a further 40% feature the loss of chromosome (Chr) 11 (7). Few genomic studies have been completed on pulmonary atypical carcinoids; however, comparative genomic hybridization studies identified recurrent deletions in Chr 11q (harboring *MEN1*) in around 60% of cases (8–10).

Tumor development is an evolutionary process with similarities to Darwinian natural selection, where tumor cells may be under multiple simultaneous “selective pressures” (11), including immune attack (12) and drug treatment (13). There are multiple debated models for the generation, propagation, and selection of variants throughout tumor development, including linear, branching, punctuated, and neutral evolution (11, 14), each of which may occur in different tumors or at different timepoints within the same patient. Tumor evolution has been associated with key clinical challenges, including cancer metastasis (15), immune evasion (12, 16), and drug resistance (13, 17, 18).

Sequencing the cell-free DNA (cfDNA) in a patient's blood plasma to identify ctDNA variants derived from tumor cells has been postulated to better represent the total disease burden (including potential tumor heterogeneity) than single tumor biopsies or resections (19, 20). However, we do not yet fully understand the detectability of ctDNA shed from different anatomic sites around a patient's body—and early results suggest that some tumor sites may be more easily detected than others (21).

In this study, we completed multimodal genomic analysis on samples collected at autopsy from 41 metastatic tumor sites from a single patient with an uncommon pulmonary atypical carcinoid, alongside a blood plasma sample and clinical biopsies, to catalog driver genomic alterations and hypothesize their order of accumulation. We compared the genomic variants identified in the tumors with those detected in the patient's blood plasma to consider how well the ctDNA analysis represented the genomic tumor heterogeneity, and in turn, infer metastatic sites that clinical ctDNA assays may poorly detect.

## Materials and Methods

### Consent, Ethical Approval, and Sample Collection

Informed written consent was obtained and a rapid research autopsy was completed in 8 hours after the patient's death under New Zealand Health and Disability Ethics committee approval 13/NTA/69/AM08, and in accordance with the Declaration of Helsinki. For a complete description of our ethical, legal, and logistical considerations, and tissue collection and sample processing protocol, please refer to Blenkiron and colleagues, 2019 (22).

### Nucleic Acid Extraction

DNA and RNA were extracted simultaneously using the Norgen formalin-fixed, paraffin-embedded (FFPE) RNA/DNA Purification Plus Kit (catalog no. 54300) from macro-dissected tumor regions of FFPE slides identified by a pathologist. DNA was extracted from blood buffy coat using the Qiagen QIAamp DNA kit (catalog no. 51304). Cell-free ctDNA was extracted from 5 mL defrosted blood plasma using the Qiagen QIAamp Circulating Nucleic Acid Kit (catalog no. 55114).

### Genomic Data Generation and Analysis

The 42 FFPE tumor samples, including 41 metastatic atypical carcinoid samples and one leiomyoma (in both cases classified by expert pathology review), were analyzed using whole-exome sequencing (WES) and transcriptome mRNA sequencing (RNA-seq). The two normal tissues were analyzed as germline controls by WES. Four tumors and one normal sample, overlapping with the WES and RNA-seq samples, were analyzed using 10X Chromium whole-genome sequencing (WGS). Clinical lung Biopsy 1 [a fine needle aspirate (FNA)], clinical subcutaneous breast Biopsy 2, and a blood plasma sample collected at autopsy were analyzed using a custom targeted DNA sequencing panel. Biopsy 1 was also analyzed by low coverage WES. All samples are described in Supplementary Table S1 and are named by the first two letters of the organ site.

DNA WES was completed as a service by Macrogen Inc., South Korea using at least 200 ng DNA per sample. Agilent SureSelect Human All Exon V6 capture (catalog no. 5190-8863) was used for 150 bp paired-end sequencing to a target depth of 200× coverage and processed through our standard exome variant calling pipeline (ref. 7; sequencing metrics in Supplementary Table S2). All variants were reviewed using Integrative Genomics Viewer (IGV, RRID:SCR_011793; ref. 23). Germline variants were curated using American College of Medical Genetics and Genomics guidelines (24). ADTEx (ref. 25; RRID:SCR_012059) was used for ploidy and zygosity estimations, while MuSiCa (26) and Signal (27) were used for mutational signature estimation, according to the tool creators’ instructions. ScarHRD was used to search for enrichment of homologous repair deficiency according to the tool creators’ instructions (28). Tumor mutational burden in mutations/Mb was calculated using the number of coding single-nucleotide variants (SNV) and indels and was compared with data from Supplementary Table S2 of Lawrence and colleagues, 2013 (29). Gene set enrichment analysis was completed using GenesetDB (30) with a FDR cutoff of 0.05. IQ-TREE (31) was used for phylogram creation from a FASTA alignment file of all variant sites, using ascertainment bias correction to account for constant sites not included in the alignment. Intertumoral heterogeneity and putative evolutionary pathways for the tumors of this patient were inferred using the LICHeE (32) and REVOLVER (33) software.

Exon target capture RNA-seq was completed as a service by Macrogen on the same tumors analyzed by DNA WES. A total of 500 ng RNA was used in the Illumina TruSeq RNA exome kit (catalog no. 20020189). Sequencing libraries underwent 100 bp paired-end sequencing with an expected output of 30 million reads/sample. Raw sequencing data were processed through our standard expression analysis and variant calling pipelines (ref. 7; sequencing metrics in Supplementary Table S3). Fusion analysis was completed using STAR-Fusion.

10x Chromium linked-read WGS was generated using high molecular weight genomic DNA extracted from fresh-frozen samples using the manufacturer's protocol (34) and the 10x Chromium Genome Library Kit & Gel Bead Kit v2 (catalog no. PN-120258). Sequencing libraries underwent 150 bp paired-end sequencing at a targeted output of over 200 Gb per sample on the Illumina HiSeq X Ten platform and were processed using the 10x “Long Ranger’ pipeline (RRID:SCR_018925, sequencing metrics in Supplementary Table S4).

A custom Thermo Fisher Scientific AmpliSeq HD panel was designed covering the 151 shared and private variants detected in WES (Supplementary Table S5). Sequencing libraries were prepared from 20 ng genomic DNA from tumor biopsies and cfDNA from blood plasma and sequenced on the Ion S5 platform with Ion 540 chip and 200 bp Ion Chef protocol (sequencing metrics in Supplementary Table S6). Data were analyzed using Ion Reporter 5.18 “AmpliSeq HD for Liquid Biopsy – w2.5 – DNA – Single Sample” pipeline with a hotspot file covering the known variant sites, and default filtering parameters.

Low-coverage WES of Biopsy 1 was completed as a service by Grafton Clinical Genomics. A total of 200 ng DNA was used in the Agilent SureSelect XT2 library kit with clinical research exome v1 capture probes, sequenced on the Illumina NextSeq500 to achieve a target of 40 million paired-end reads and processed through our standard exome variant calling pipeline (ref. 7; sequencing metrics in Supplementary Table S7).

### Data Availability Statement

Access to the datasets generated in this study is controlled out of respect for the deceased patient, however, may be requested through by a data management committee, made available from the European Genome-phenome Archive (https://www.ebi.ac.uk/ega/home; accession number EGAS00001006530).

## Results

### Clinical Summary and Overview of Tissue Samples

**FIGURE 1:**
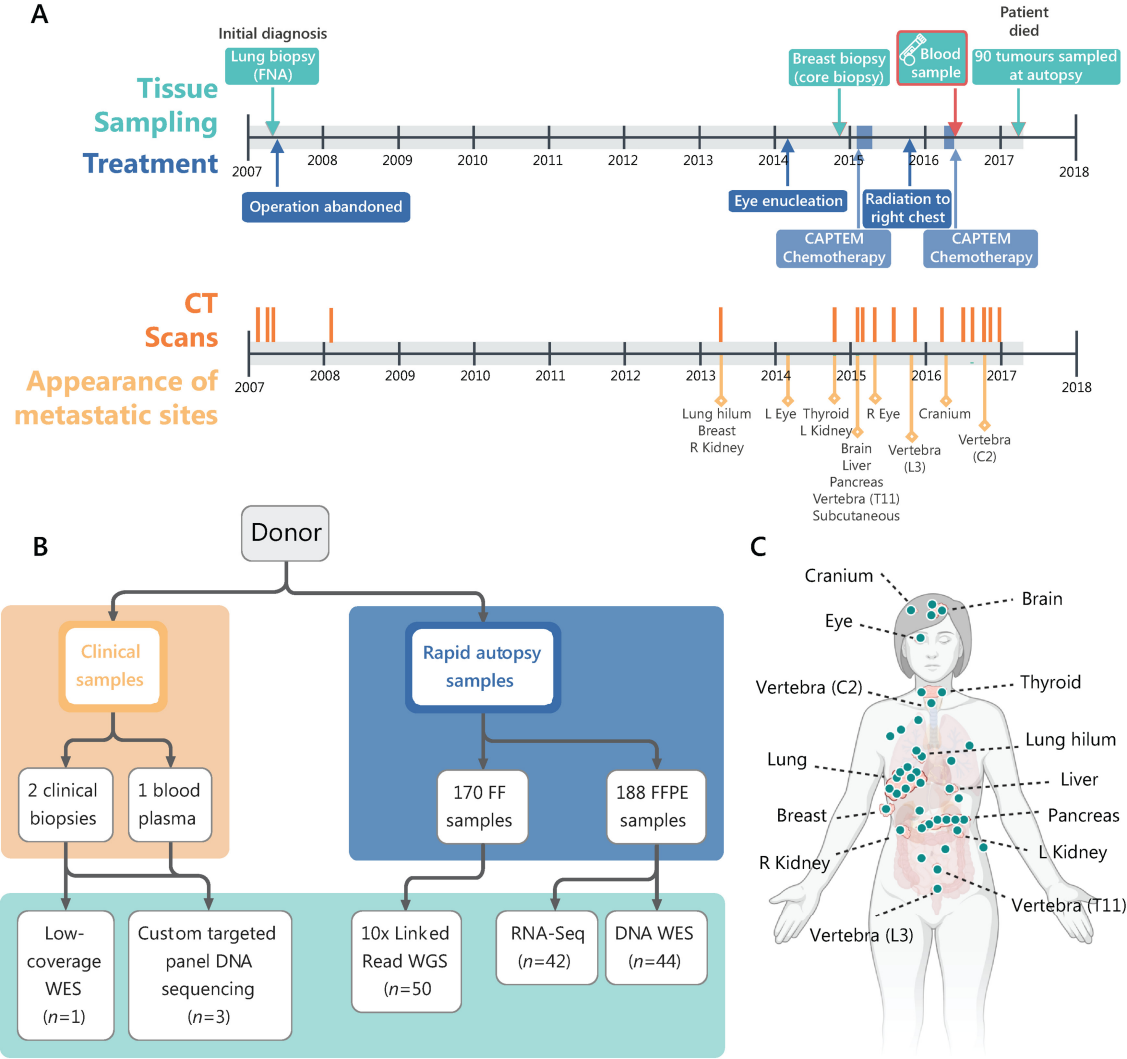
Clinical summary and overview of tissue samples included in this study. **A,** Timeline of tissue sampling, treatments, imaging, and appearance of metastatic sites. Top panel shows tissue sampling (turquoise, including biopsies taken as part of clinical care, blood sample, and autopsy sampling) and treatment (blue). Bottom panel displays dates of CT scans (orange) and details the first appearance of metastatic sites within those CT scans (yellow). Arterial involvement meant that the cancer was not resectable at initial diagnosis. There was minimal clinical follow-up for 7 years after initial diagnosis. After presenting with reduced vision in her left eye, caused by a metastasis, CT scans showed marked growth of the lung primary and metastases to thyroid, kidney, and breast. A year later, further lesions were detected in the brain, kidney, liver, pancreas, and numerous subcutaneous sites. Capecitabine-temozolomide (CAPTEM) combination chemotherapy was administered, stabilizing growth in most lesions, alongside a short course of radiotherapy to the hilum of her right lung. In 2016, she began losing sight in her remaining right eye, with further progression of the brain lesion noted on CT imaging. Further chemotherapy was administered with a clinical response. The patient died in April 2017, and the rapid research autopsy was completed within hours of her death. **B,** Clinical and autopsy samples collected and their downstream genomic data applications. **C,** Locations of the 41 metastatic sites sampled at autopsy that were included in this study. Further samples not labeled were located within the subcutaneous tissue. Figure 1C created with BioRender.

A 69-year-old woman who had never smoked was diagnosed in 2007 with an atypical carcinoid tumor at the base of her right lung. Surgical resection was attempted but abandoned because of significant pulmonary artery involvement. When she died 10 years later, the tumor had spread to 90 anatomic locations detected on imaging, including brain, eyes, thyroid, liver, pancreas, kidneys, vertebrae, cranium, and many subcutaneous sites (clinical course summarized in Fig. 1A). A rapid research autopsy was completed within 8 hours after her death. A total of 358 tissue samples were collected from the 90 tumors. All tumors were histologically similar; therefore, a representative subset of tumors to be analyzed genomically was selected to include a diverse range of widely separated anatomic sites. We selected at least one sample of all large metastatic foci, and selected multiple samples for especially large lesions (16 cm^3^) to allow investigations of intratumor heterogeneity. A total of 42 tumor samples (including one uterine leiomyoma) and two non-tumor samples (heart muscle and blood buffy coat) underwent multiple genomic analyses (Fig. 1B). In addition, we included two tumor biopsies taken as part of clinical care (primary lung tumor FNA at diagnosis and breast tumor biopsy) and one blood plasma sample taken as part of a research project ten months before the patient passed away.

### Genomic Landscape of Chromosomal Alterations

**FIGURE 2:**
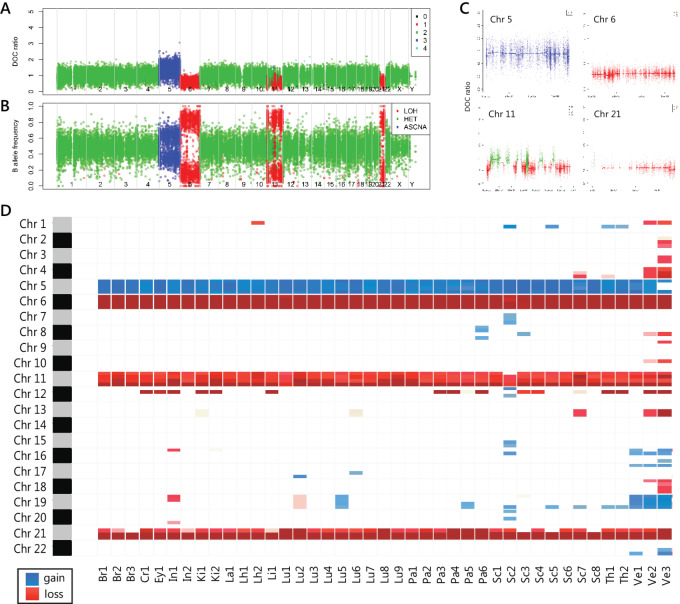
Most chromosomal alterations are shared by all tumors. ADTEx copy-number profile of representative tumor sample Lu2 (lung primary) showing the chromosomal alterations shared by all tumors. **A,** Normalized depth of coverage (DOC) ratio between tumor and normal samples at SNVs for all chromosomes, separated by vertical lines. Color represents predicted copy number—red = 1 copy (loss), green = 2 copies (diploid), blue = 3 copies (gain). **B,** B-allele frequency (BAF) of SNVs. The BAF of SNVs on Chr 5 fall around 0.33 and 0.66, in line with the gain of one copy of Chr 5. BAF approach 1 and 0 in regions of Chr 6, 11, and 21, where one copy is lost. Abbreviations in key to right: LOH, loss of heterozygosity; HET, diploid heterozygous; ASCNA, allele-specific copy-number amplifications. **C,** Normalized DOC ratio for all chromosomes where shared chromosomal alterations were predicted in all tumors: Chr 5 gain, Chr 6 loss, oscillating pattern in Chr 11 with alternate regions lost, and Chr 21 loss. **D,** CNVs summarized across whole tumor cohort, with data summarized at the half arm level, showing common alterations to Chrs 5, 6, 11, and 21. Increased predicted CNVs in vertebral samples (Ve1,2,3) were likely related to poor-quality DNA resulting from decalcification.

WES was performed on 41 metastatic atypical carcinoid samples, one leiomyoma and two normal samples (Fig. 1C). The benign uterine leiomyoma (Ut1) was, as expected, genomically distinct from the metastatic atypical carcinoid samples, so it will not be discussed further. Somatic chromosomal alterations were inferred from WES using ADTEx, revealing that all metastases sampled at autopsy shared the gain of one copy of Chr 5, loss of one copy of Chr 6, a complex “shattering” event of one copy of Chr 11 and loss of one copy of Chr 21 (Fig. 2). Biopsy 1 (diagnostic primary lung tumor FNA sampled 10 years earlier) featured the gain of Chr 5 and loss of Chr 21; however, there was insufficient evidence to definitively confirm the presence of changes to Chr 6 and Chr 11 in this historical biopsy (Supplementary Fig. S1) due to limited DNA mass and data quality (10.8 million reads were generated, resulting in only 15% of the exome footprint being covered with >30× coverage). The loss of one copy of a 246 kb region of Chr 12p was found in 15 of 41 tumors sampled at autopsy (Fig. 2D), disrupting *ETV6* and resulting in loss of heterozygosity (LOH) of 360 genes, including tumor suppressor *CDKN1B*.

### Chromothripsis Breakpoints Revealed by Linked-read Sequencing

**FIGURE 3:**
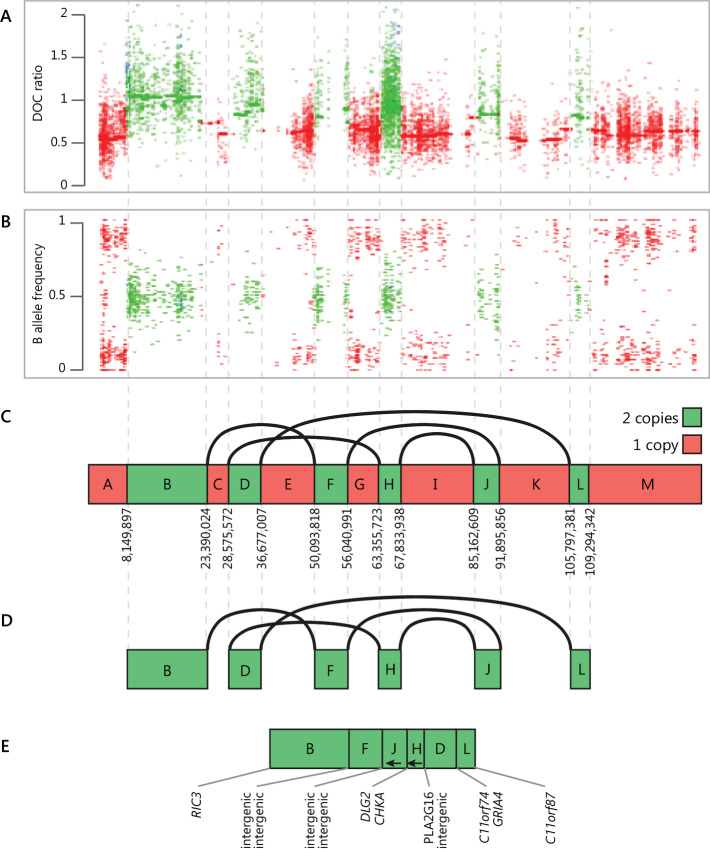
Chromothripsis of Chr 11 determined by 10× linked-read WGS. The rearranged chromosome contains two copy-number states (regions in red are lost while remaining green regions are “stitched together” in a different order). **A** and **B,** WES-based normalized DOC ratio and BAF from ADTEx, of insufficient resolution to resolve structural complexities. **C,** Precise chromosomal breakpoints identified using linked-read WGS with inferred copy number of each region. **D,** The altered Chr 11 results from five breakpoint joining events. **E,** Altered Chr 11, indicating the inversion of two regions (**J** and **H**, indicated with arrows) and deletion of the regions A, C, E, G, I, K, and M. The genes at each breakpoint boundary are indicated below the chromosome. The wild-type copy of Chr 11, which contributes to **A–C** is not shown in **D** and **E**, which illustrate only the altered copy.

A feature of all tumors sampled at autopsy was the “shattered” Chr 11, indicative of chromothripsis. The precise junctions of 12 breakpoints on Chr 11 were not unequivocally identified by short-read sequence data but were revealed by linked-read sequencing generated on four representative tumor samples in comparison with one normal sample. Seven regions were lost from one allele of Chr 11, and the six remaining regions were reassembled in a new order, while the other allele of Chr 11 was unaffected (Fig. 3, with evidence of each breakpoint in Supplementary Fig. S2). Genes *PLA2G16, RIC3*, and *C11orf87* were disrupted, and two possible fusion gene products were created (*DLG2-CHKA* and *C11orf74-GRIA4*; Fig. 3), while all other breakpoints fell in intergenic regions. The *DLG2-CHKA* fusion involved joining the first three (of 42) exons of *DLGA* to exons 8–14 of *CHKA*. It was absent from STAR-Fusion RNA-seq fusion analysis completed on the same tumor samples, suggesting the transcript may be reduced in abundance by nonsense-mediated decay. The *C11orf74-GRIA4* fusion joined the last 12 exons of *GRIA4* to the end of *C11orf74* after seven (of eight) exons and was transcribed and detected in the RNA-seq in most tumors, with premature truncation shortly after the breakpoint. The lost regions of Chr 11 included the locus containing *KMT2A*, a frequent site of LOH in NETs (35), and gene set enrichment analysis of the genes lost revealed significant enrichment for the olfactory signaling, matrix metalloproteinases, and folate metabolism gene sets (Supplementary Table S8).

### Genomic Landscape of Small Variants

**FIGURE 4:**
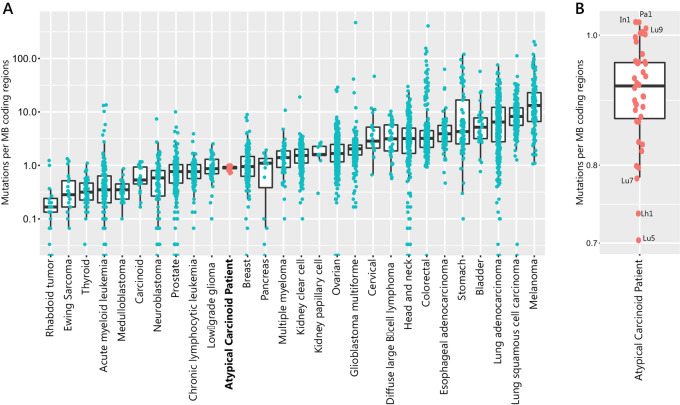
Overall mutational burden is low compared with other tumor types. **A,** TMB in lung NET patient tumors sampled at autopsy (pink) compared with other cancer types analyzed in Lawrence and colleagues 2013 (29). *Y*-axis is on a log scale (mutations per MB). **B,** Expanded diagram showing TMB across tumors collected at autopsy from atypical carcinoid patient, with highest and lowest three tumors labeled. *Y*-axis is on a linear scale.

No pathogenic or likely pathogenic germline variants were detected, consistent with the patient's diagnosis in her seventies with no known family history of cancer. Tumor mutational burden (TMB) was around 1 variant per coding MB, in the lower third of TMB when compared with other well-studied cancer types (ref. 29; Fig. 4A). Our patient's tumors featured relatively consistent somatic TMB, ranging from 0.7 mutations per MB for some primary lung tumor samples (which may be representative of an earlier genomic state) to around 1 for Pa1, In1, and Lu9 (pancreas, abdominal wall nodule, and lung; Fig. 4B). The mutational signature making the greatest contribution was Signature 3, associated with *BRCA1/BRCA2* inactivation (Supplementary Fig. S3A); however, it was only significantly enriched in two tumor samples [abdominal wall nodule In2 (60%) and pancreatic metastasis Pa2 (41%)] and did not reach the threshold for statistical significance in other samples. There were no *BRCA1/BRCA2* or other homologous recombination DNA repair–related gene mutations identified in the WES, despite adequate depth of coverage. No other mutational signatures were significantly enriched. To robustly exclude homologous recombination DNA repair deficiency in this patient's metastatic tumors, we analyzed exome data from the tumors using the scarHRD package (28). All tumors, including the two that had a significantly enriched mutational signature 3 contribution (In2 and Pa2), had an HRD-sum score < 20; whereas known HR-deficient tumors invariably have HRD-sum scores of > 40 (ref. 28; Supplementary Fig. S3B).

**FIGURE 5:**
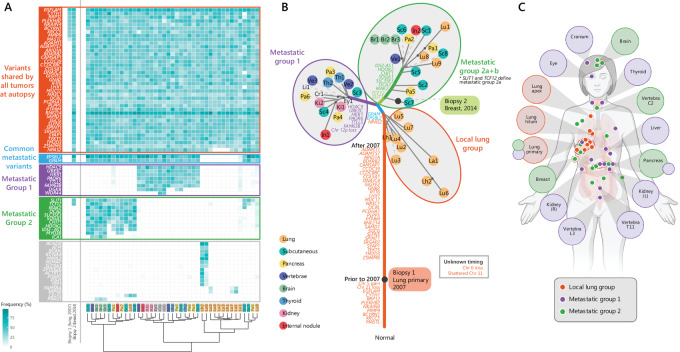
Genomic similarity of tumors around the patient's body. **A,** Variant allele frequency (VAF) of variants present in two or more samples. Variants are named by gene, presented in rows, with all variants regardless of pathogenicity or protein effect presented. Tumors are presented in columns, with clinical biopsy samples on the left (biopsy 1, collected from lung primary at first diagnosis in 2007, and biopsy 2, collected from breast metastasis in 2014) and samples collected at autopsy on the right (colored by organ, key to right). Boxes are shaded according to VAF (key bottom left). Bottom panel features Ward's hierarchical clustering dendrogram grouping similar tumors (Euclidean distance). Variants are grouped into blocks based on the tumors carrying each variant, with coloring consistent across figures. *SLIT1* and *TCF12* variants in metastatic group 2 are present in more samples than the remainder, so are defined as metastatic group 2a in Fig. 6. **B,** DNA phylogram of clinical biopsies alongside tumors sampled at autopsy, with branch length proportional to the number of shared small variants, constructed in IQ-TREE (31) using the K2P substitution model. Small variants and chromosomal alterations are annotated on the branches. Sample coloring indicates major anatomic locations (key to left, shared with **A**). Gray ovals group samples into three major tumor groups based on variant profiles. While the order of accumulation of groups of variants can be inferred from this analysis, it does not suggest the accumulation order within groups. Dotted lines are used in some cases to indicate the position of tumors and ensure all labels are readable. Large black dots indicate the placement of biopsy samples. Shared small variants and copy-number changes are placed according to presence or absence in Biopsy 1. The patterns of relatedness derived from the small variant data were consistent with the patterns in chromosomal variants; however, alone they do not provide the fine-grained separation visible from the small variants. **C,** Anatomic location of genomic groups. Colored circles overlaid on body represent tumor sampling sites colored according to the genomic group(s) present. Tumors are labeled and shaded according to dominant genomic group(s). Unlabeled sites were located in subcutaneous tissue. Figure 5C created with BioRender.

All tumors sampled at autopsy shared 39 small variants (Fig. 5A). None of these shared variants were found in ClinVar, COSMIC or The Cancer Genome Atlas, and only a heterozygous variant in *ARID1A* was predicted to be a driver by the Cancer Genome Interpreter (CGI) algorithm (ref. 36; Supplementary Table S9). This single base pair deletion [chr1:g.27100383del, p.(Q1365Hfs*116)] in the “hypermethylated in cancer” domain of exon 17 causes a frameshift at amino acid residue 1365, leading to premature protein truncation.

A custom Thermo Fisher AmpliSeq HD sequencing panel covering all variants identified in tumors sampled at autopsy was used to interrogate the two clinical biopsy samples. Of the 39 variants shared by all tumors sampled at autopsy, just nine were detected in Biopsy 1 (diagnostic biopsy of lung primary tumor, 2007), sampled before there was evidence of metastatic disease by imaging or clinical examination, and are assumed to have accumulated earliest in tumor development (Fig. 5B). Of these, only three variants were predicted to be protein altering (in *RRP12*, *MMP9,* and *MASTL*; Supplementary Table S10); however, there is no evidence that these variants are associated with tumor development (36, 37). Those not detected in Biopsy 1 were sufficiently covered in the sequencing data to call as unequivocally absent in that biopsy (depth ranged between 1,065 and 9,463 unique DNA molecules). Surprisingly, the *ARID1A* putative driver variant was not detected despite the variant site being covered by 7,240 unique DNA molecules, so it likely developed after the initial diagnosis (Supplementary Fig. S4). Therefore, the gain of one copy of Chr 5 and the loss of one copy of Chr 21 (identified from low-coverage WES of Biopsy 1) preceded the development of the *ARID1A* variant.

A subset of samples from within the lung tumor carried very few additional variants over the 39 shared variants found in all lesions at autopsy (e.g., Lu5 and Lu7; Fig. 5B) and may possibly represent the most genomically similar tumors to the patient's historical primary lung tumor. Two variants were identified in all tumor samples situated outside the lung and three lung samples (*GPAM* and *EPS8L2*; Fig. 5A and B). Visual inspection of the sequencing data in IGV confirmed the absence of these variants in the remaining nine lung samples, despite adequate sequencing depth. Two further genomic groups were identified in the metastatic tumors sampled at autopsy. “Metastatic group 1,” identified by nine variants, spread to multiple vertebrae, the pancreas, thyroid, both kidneys, cranium, liver, subcutaneous sites, eye, and was also found in one sample from the lung tumor (Lu1, shown in group 2 in Fig. 5B due to subclonality). “Metastatic group 2,” identified by 11 variants, was detected in subcutaneous sites, pancreas, brain, vertebra and was also found in the lung tumor. Because all major genomic groups were represented within the lung tumors (Lu1 in “metastatic group 1,” Lu8 and Lu9 in “metastatic group 2,” apparent in Fig. 5C) it is possible that the metastatic clones may have arisen from this location. Within-sample genomic heterogeneity was evident in lung and pancreatic samples Pa1, Lu1, Lu8 and to a lesser extent Pa5, with evidence of both metastatic clones intermixed in these samples.

Biopsy 2 (subcutaneous breast, 2014) carried the same variants as sample Sc7 (from the same anatomic site sampled at autopsy, 2017), indicating that this lesion did not develop further genomic alterations in the 3 years before the patient died and suggesting that it remained genomically stable, despite its large size (19 cm^3^). Two rounds of chemotherapy were given in the interval between these two samples being taken (Fig. 1A); however, no additional variants consistent with selection driven by chemotherapy were identified.

Apart from the two clinical biopsies, all other samples were collected at a single timepoint (at autopsy). Nevertheless, we were able to apply two bioinformatic methods to this DNA dataset to first explore putative tumor phylogeny and subclonal structure (LICHeE) and secondly to infer the possible trajectory of variant acquisition (REVOLVER). The putative subclonal structure and evolutionary trajectory that appear most likely given this patient's metastatic genomic landscape are shown in Supplementary Fig. S5.

### Representing Tumor Heterogeneity in ctDNA

Having cataloged the shared and private small somatic variants held by this patient's sequenced tumors, we identified those detectable in a blood plasma cfDNA sample taken 10 months before the patient died. A custom Thermo Fisher Scientific AmpliSeq HD panel covering all the shared and private variants identified in tumors sampled at autopsy (Supplementary Table S5) was used for ctDNA sequencing, achieving a median depth of coverage greater than 6,000 unique molecules across variant sites.

**FIGURE 6:**
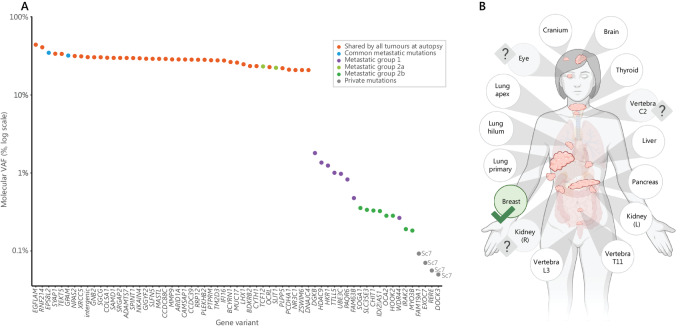
Origins of detected ctDNA. **A,** Molecular VAF of tumor variants detected in the blood plasma. Variants are colored by their “shared” status (consistent with Fig. 5, key to right). Molecular VAF (%) is plotted on a log scale. Private variants are labeled with the sample name. Variants shared by all tumors are detected at the highest molecular VAF. **B,** Ability to detect ctDNA from different organ sites based on the detection of one or more private variants in ctDNA with more than three variant molecules, indicated with green tick. Three tumors did not carry any private variants, so we were unable to uniquely determine whether they shed ctDNA into the blood plasma using this assay (eye, vertebra C2 and right kidney, indicated with question marks). The only private variants detected were derived from a large subcutaneous breast sample Sc7. Figure 6B created with BioRender.

The variants detected at the highest frequency in the ctDNA were shared by all tumors and, by inference, present in all cancer cells in the patient's body (at 20%–40% variant molecules; Fig. 6A). Two variants identifying metastatic group 2a (*SLIT1* and *TCF12*) were detected at a similarly high level (around 20% variant molecules). Variants from metastatic groups 1 and 2b were detected at a markedly lower level (at 1%–3% variant molecules and 0.2%–0.6% variant molecules, respectively).

We searched for the presence of private variants, unique to each sampled site, in ctDNA to infer whether that lesion was uniquely detectable in the blood plasma, given that private variants would be shed from a single tumor site. (Fig. 6B). The only private variants detected were derived from subcutaneous breast sample Sc7 (four variants, each detected with 5–8 unique molecules), suggesting this may be a clear site of peripherally detected ctDNA release. No other private variants were detected with more than three unique molecules [the manufacturer's recommended cutoff for reporting variants (38), explored in Supplementary Fig. S6].

## Discussion

This study investigated a large number of metastatic tumors (two clinical biopsies, 42 tumor samples collected at autopsy and a blood sample collected during patient care) in a single patient with pulmonary atypical carcinoid cancer. Our aim was to provide a high-resolution understanding of the heterogeneity and putative developmental trajectory of this uncommon cancer type, complementing studies of more common cancer types that often involve large numbers of patients each with relatively small numbers of metastatic tumors. While a range of small variants and copy-number alterations have been reported previously in pulmonary atypical carcinoid tumors (9, 10, 39–41), no studies have explored the heterogeneity and relative timing of accumulation of multiple types of genomic alterations in this tumor's development.

### Somatic Cancer Driver Variants

No pathogenic germline variants were detected, and while the diagnostic biopsy contained numerous small variants not found in the patient's germline, none of these were predicted to be oncogenic drivers. However, large-scale chromosomal changes were present in the diagnostic biopsy and all other tumors of this patient (gain of one copy of Chr 5, loss of one copy of Chr 21). Given the clear absence of other driver mutations, we hypothesize that these chromosomal changes are likely to have driven, or at least strongly contributed to, initial tumor development. This chromosome 5 amplification is consistent with previous reports in atypical carcinoid tumors of recurrent gene-level copy-number changes on the p arm of Chr 5 (40).

In addition, all metastatic tumors collected at autopsy had loss of one copy of Chr 6 and chromothripsis of one copy of Chr 11. While the precise Chr 11 chromothripsis event described here is not reported in other studies, one atypical carcinoid cancer has been described with chromothripsis of Chr 11 and Chr 20 (42), and Chr 11 chromothripsis has been reported in other types of NET (43). In addition, deletions on the q arm of Chr 11 have been reported in between 50% and 72% of atypical carcinoid tumors (8–10), consistent with the general disruption of genes on chromosome 11 in this tumor type.

All metastatic tumors collected at autopsy also contained numerous small somatic variants not found in the diagnostic biopsy. However, only one of these small variants was predicted to drive tumor development; a heterozygous *ARID1A* variant. Somatic *ARID1A* variants, often heterozygous (44), have previously been found in atypical carcinoid tumors (6, 39) and have also been implicated in the pathogenesis of numerous other cancer types due to the role of ARID1A in the SWI/SNF chromatin remodeling complex (45), where *ARID1A* variants cause increased cell proliferation, migration, and invasion through a variety of mechanisms (46). The absence of the *ARID1A* variant in the diagnostic lung biopsy suggests that it most likely drove later tumor progression and was not an initiating driver variant, despite being identified as a driver in other cancer types and being recurrently detected in previous lung carcinoid studies (6, 39).

### Models of Tumor Evolution and Metastatic Dissemination

**FIGURE 7:**
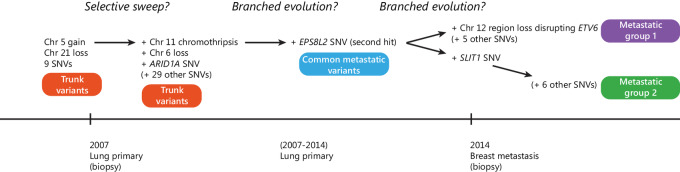
Hypothetical somatic driver variant accumulation over time and space based on the genomic data. A selective sweep may have occurred to sweep to fixation the variants found in every tumor sample sequenced at autopsy, but not present in the initial lung tumor biopsy. Branching events likely occurred subsequently to generate the tumor heterogeneity detected in the tumors sequenced at autopsy.

We can hypothesize a putative genomic evolution process (Fig. 7) that is consistent with the genomic heterogeneity observed at autopsy and in the two clinical biopsies. The early lung tumor represented by the diagnostic biopsy featured gain of Chr 5, loss of Chr 21 as well as nine small variants, none of which are known or predicted cancer drivers. Genomic features of the tumors collected at autopsy are consistent with further mutation after the lung biopsy was undertaken, followed by an evolutionary event such as a selective sweep, that fixed into the genomes of subsequent tumors a loss of function variant in *ARID1A* as well as 29 small non-driver variants and additional chromosomal alterations including Chr 11 chromothripsis and Chr 6 loss. There are plausible selective advantages to the tumor of each, including Chr 6 LOH of the HLA genes responsible for presenting peptides to T cells, hypothetically reducing the immune system's ability to recognize neoantigens presented by the tumor cells (47). The data are consistent with subsequent branching events, including the accumulation of a second somatic “hit” to *EPS8L2* (where the first was somatic LOH on Chr 11), before two parallel lineages developed, metastatic group 1 and 2 (Fig. 7). In metastatic group 1, some variants of unknown significance deserve further study. For instance, truncation of tumor suppressor *ETV6* [a common fusion gene partner in breast and thyroid cancer (48)], following the LOH of a region of Chr 12 may be functionally significant given this gene's role in development (49). The missense *SLIT1* variant was the only additional protein-affecting variant carried by tumors in metastatic group 2 and may play a role in angiogenesis and migration (50). Interestingly, the two metastatic groups in the pancreas appear to have arisen by two independent seeding events. However, in the literature there is not overwhelming evidence for consistent genetic drivers of metastatic progression (51, 52) and we have no evidence in our data to suggest the additional genomic variants accumulated by each of the two metastatic groups (e.g., in *EPS8L2*, *ETV6,* and *SLIT1*) were necessary to drive their metastasis. Given a general propensity of cancers to metastasize to lung (53), it is possible that the presence of multiple metastatic groups in the lung may be the result of tertiary metastatic seeding events returning tumor cells that have evolved elsewhere to the site of the lung primary tumor.

The most likely subclonal structure and mutational progression hypothesized by the LICHeE and REVOLVER methods are consistent with the simple clustering analysis and phylograms shown in Fig. 5. While this consistency is interesting, we agree with the widely held opinion that inference of evolutionary history based on single timepoint data should be considered hypothetical (54).

### What Does the Sequencing of ctDNA Reveal?

ctDNA analysis is emerging as a valuable minimally invasive clinical test for patients with cancer (55, 56), capable of identifying the evolution of resistance mutations to targeted therapy (57). Here, we had an opportunity to compare the defined solid tumor DNA variant burden with that detectable circulating in the patient's blood plasma. The variants most readily detectable were shared by all or most tumor cells. The overall genomic heterogeneity was clearly detectable, with higher frequency variants being common to all or most lesions and lower frequency variants representing evolutionary outcrops. In the clinical setting where ctDNA sequencing would likely be completed without extensive solid tumor sequencing, ctDNA analysis would have identified the truncal variants that are shared by all metastases. While some other past studies have compared tumor variants identified in metastatic solid tumors and ctDNA (58, 59), few have investigated differences in the detection of ctDNA between anatomic sites. In this study, only the private variants in a single metastasis, breast lesion Sc7, was detected in this patient despite the larger size of other tumors including lung, thyroid, and pancreas (Fig. 6). The detection of ctDNA in the blood plasma derived from localized brain, thyroid, pancreas, and kidney tumors has been poor in some previous studies (21, 60, 61), as in our study, suggesting fundamental barriers to ctDNA detection including the blood–brain barrier, mucinous features (21), tumor desmoplasia (62) and first-pass metabolism by the liver (21). However, a past study in melanoma has identified that subcutaneous disease was not well represented in the ctDNA (63), in contrast to our finding. This suggests that the use of ctDNA for early detection of small treatment-resistant subclones may be challenging until more sensitive methods are developed and evaluated, especially if these subclones evolve in tissue types known to export little ctDNA (21, 60–62).

### Study Limitations

We acknowledge that studies that analyze many tumors from a single patient, while revealing detailed and valuable insights about that patient's cancer, cannot necessarily be generalized to other patients. Furthermore, all samples except for two clinical biopsies were collected at a single timepoint (at autopsy), limiting the extent of computational tumor evolution inferences that could be made, and the small size of these biopsies limited the scope of genomic assays that could be applied. Unfortunately, tissue of the two clinical biopsies was significantly depleted after clinical standard-of-care testing. This precluded additional analysis such as expanded targeted sequencing, which could more specifically place these samples in the evolutionary cascade of this patient's tumors. Furthermore, while RNA-seq data from this patient's tumors were useful for confirmation of expressed mutations and for identification of fusion gene products, a complex batch effect attributable to tissue processing artefacts in the autopsy samples could not be corrected using statistical approaches (Supplementary Table S3). This precluded the use of the RNA expression data in techniques such as molecular pathway analysis and differential expression analysis. Functional studies investigating the role of variants of uncertain significance, including chromosomal aberrations, were outside the scope of this study but provide an important avenue of future research.

In summary, DNA sequencing data generated from 42 widespread tumor samples, alongside clinical biopsies and blood plasma, have provided a high-resolution understanding of the genomic heterogeneity and putative evolution of this uncommon cancer type. In particular, this study provides evidence of the critical early role that chromosomal alterations may play in atypical carcinoid tumor progression. ctDNA analysis readily identified truncal variants but was unable to detect the private variants of most tumor subclones, despite high depth of coverage. This has implications for the use of ctDNA in cancer evolution studies and encourages further evaluation in expanded patient cohorts. We are grateful to the patient and her family for their contribution to scientific research.

## Supplementary Material

Supplementary Tables S1-S10 Supplementary Table S1 includes a description of the tumour samples analysed.
Supplementary Table S2 includes DNA WES summary statistics.
Supplementary Table S3 includes RNA-Seq summary statistics.
Supplementary Table S4 includes WGS linked-read summary statistics.
Supplementary Table S5 includes regions covered in the custom ThermoFisher Scientific Ampliseq HD panel. Supplementary Table S6 includes targeted DNA sequencing custom panel summary statistics. Supplementary Table S7 includes low-coverage DNA WES summary statistics.
Supplementary Table S8 includes gene set enrichment of genes lost from Chr 11.
Supplementary Table S9 includes variants identified in two or more tumour sites.
Supplementary Table S10 includes the consequence of somatic variants in Biopsy 1.

Supplementary Figure S1 Supplementary Figure S1: ADTEx copy number analysis of Biopsy 1 from low coverage WES Copy number profile of low-coverage WES of Biopsy 1 revealing A, likely amplification of Chr 5 and B, loss of Chr 21. Top panels display DOC ratio, colored by predicted copy number state. Bottom panels display BAF, colored by predicted copy number alteration. The separation of BAF towards 0.3 and 0.6 in Chr 5 indicates chromosomal gain, whereas the separation of BAF towards 0 and 1 in Chr 21 indicates LoH. There was insufficient evidence conclusively identify the presence or absence of the loss of C, Chr 6 and D, chromothripsis of one copy of Chr 11 based on ADTEx analysis of the low-coverage WES of Biopsy 1, however it is likely that they were absent. Copy number analysis was plagued with high levels of noise from low coverage and large differences in coverage between the Biopsy 1 and normal sample. The sole Chr 11 breakpoint covered by the exome sequencing did not provide read support for chromothripsis. The apparent amplifications of regions of each chromosome do not match up to any known alterations in high-quality DNA samples from tumours collected at autopsy and are likely attributable to noise in the low coverage WES.

Supplementary Figure S2 Supplementary Figure S2: Breakpoints in 10x Loupe software linked-reads view.
Each bar represents a sequencing read, and those joined with a horizontal line share the same barcode.
Reads are grouped by haplotype (green and purple), with unphased reads in grey. Vertical orange lines indicate putative breakpoints defined by the software. Given that the maximum region of the chromosome phased was significantly shorter than the length of Chr 11 (as indicated in the ‘phase block view’ plot), some breakpoints are assigned to different haplotypes, therefore haplotypes should not be compared between regions (the breakpoint appears in purple in some regions and in green in others).
The copy number patterns are consistent with all breakpoints occurring on the same copy of the chromosome.

Supplementary Figure S3 Supplementary Figure S3: Mutational signatures across all samples collected at autopsy A, Overall mutational burden does not reveal significant mutational signatures (MuSiCa). The signature contribution is indicated by the degree of shading (key to right). The signature with the highest contribution was Signature 3, however it was not statistically significant in most samples according to tool Signal. B, The scarHRD method for quantifying homologous recombination deficiency did not identify any tumors to be HR-deficient; with all having an HRD-sum score < 20 (known HR-deficient tumors usually have HRD-sums of > 40)28.

Supplementary Figure S4 Supplementary Figure S4: Absence of ARID1A variant in targeted panel sequencing of Biopsy 1 despite adequate depth Top panel shows Biopsy 1 targeted panel sequencing. Bottom panel shows representative tumor sampled at autopsy (Sc6) with heterozygous deletion in ARID1A, clearly absent from Biopsy 1 sequencing despite adequate depth (7240 unique molecules). The probability of not sampling the ARID1A variant in Biopsy 1 due to chance alone was calculated assuming tumor cellularity of 80% and the heterozygous ARID1A variant being present in 50% of tumor reads. 7240 unique molecules covered this genomic position. The binomial distribution in R was used to calculate the probability of not sampling this variant: dbinom(7240, size=7240, prob=0.6).

Supplementary Figure S5 Supplementary Figure S5: Inference of putative sequences of genomic changes consistent with the data using the LICHeE and REVOLVER methods LICHeE and Revolver were used to further speculate the order of genomic changes in this patient's dataset. ClonEvol was also trialed, using PyClone putative clonal clusters as input, however the results did not converge. A, LICHeE was run according to the tool creator's instructions, using VAFs as input, to generate clonal relationships of tumor samples. Numbers inside colored circles represent the number of mutations defining a clone, and the squares represent the clonal structures of individual samples. Shaded regions indicate the proportion of cells belonging to that clone, where the white regions represent normal cells. Four dominant tumor groups were apparent. Where two or more colors are present, LICHeE has predicted the sample to contain a mixture of clones (most apparent in Pa1 and Lu1 but also present in In2, Pa6 and others). This feature is also visible in the VAF plot (Figure 5a) but not represented on the DNA phylogram (Figure 5b). Technical reasons prevented the inclusion of some samples, e.g. Lu8 and Pa5. B, Clonal relationships between tumor samples as predicted by REVOLVER, using PyClone putative clonal clusters as input (from VAF and chromosomal copy number), and default tool parameters. Clones are labeled according to one variant defining that clone (e.g., MASTL), and the circle size is proportional to the number of variants defining the clone. Number refers to clone number. REVOLVER highlighted the same pattern of progression seen in other analyses: from normal cells, common variants accumulate (labeled as MASTL in Figure 5b), and a further set of variants shared by most tumors was identified (labeled GPAM) before a split into two dominant tumor groups, each characterized by their own set of variants (labeled SLIT1 and PAQR6 respectively). REVOLVER highlights the progression of variant accumulation across all samples, rather than indicating individual samples. All coloring consistent with Figure 5. Overall, LICHeE and REVOLVER explore the clonal and subclonal structure of individual tumor samples and suggest the same sequence of genomic progression evident in the DNA phylogram (Figure 5b). None of these cancer-specific methods attempts to time the evolutionary divergence events.

Supplementary Figure S6 Supplementary Figure S6: ctDNA detection of private variants While only four private variants were reported by the sequencing platform’s IonReporter software (indicated in orange, key top right), there were sequencing reads to support other private variants. Private variants unique to samples La1 (3 variants) and Pa6 (1 variant) were supported by 2 to 3 variant molecules, and there was a single variant molecule detected to support private variant detection in the following samples: Br2, Cr1, In2, Ki2, La1, Lh1, Lh2, Lu6, Lu7, Lu9, Pa2, Pa3, Pa6, Sc7, Th1, Th2, Ut1, Ve2, Ve3.
