# Supplementary material for: Complex Patterns of Genomic Heterogeneity Identified in 42 Tumor Samples and ctDNA of a Pulmonary Atypical Carcinoid Patient
Source: Cancer Res Commun. 2023 Jan 10;3(1):31–42. doi: 10.1158/2767-9764.CRC-22-0101 (PMC10035512; doi:10.1158/2767-9764.CRC-22-0101)
Supplement: Supplementary Figure S1 — ADTEx copy number analysis of Biopsy 1 from low coverage WES Copy number profile of low-coverage WES of Biopsy 1 revealing A, likely amplification of Chr 5 and B, loss of Chr 21. Top panels display DOC ratio, colored by predicted copy number state. Bottom panels display BAF, colored by predicted copy number alteration. The separation of BAF towards 0.3 and 0.6 in Chr 5 indicates chromosomal gain, whereas the separation of BAF towards 0 and 1 in Chr 21 indicates LoH. There was insufficient evidence conclusively identify the presence or absence of the loss of C, Chr 6 and D, chromothripsis of one copy of Chr 11 based on ADTEx analysis of the low-coverage WES of Biopsy 1, however it is likely that they were absent. Copy number analysis was plagued with high levels of noise from low coverage and large differences in coverage between the Biopsy 1 and normal sample. The sole Chr 11 breakpoint covered by the exome sequencing did not provide read support for chromothripsis. The apparent amplifications of regions of each chromosome do not match up to any known alterations in high-quality DNA samples from tumours collected at autopsy and are likely attributable to noise in the low coverage WES. [file crc-22-0101-s02.pdf]

Supplementary Figures S1-S6

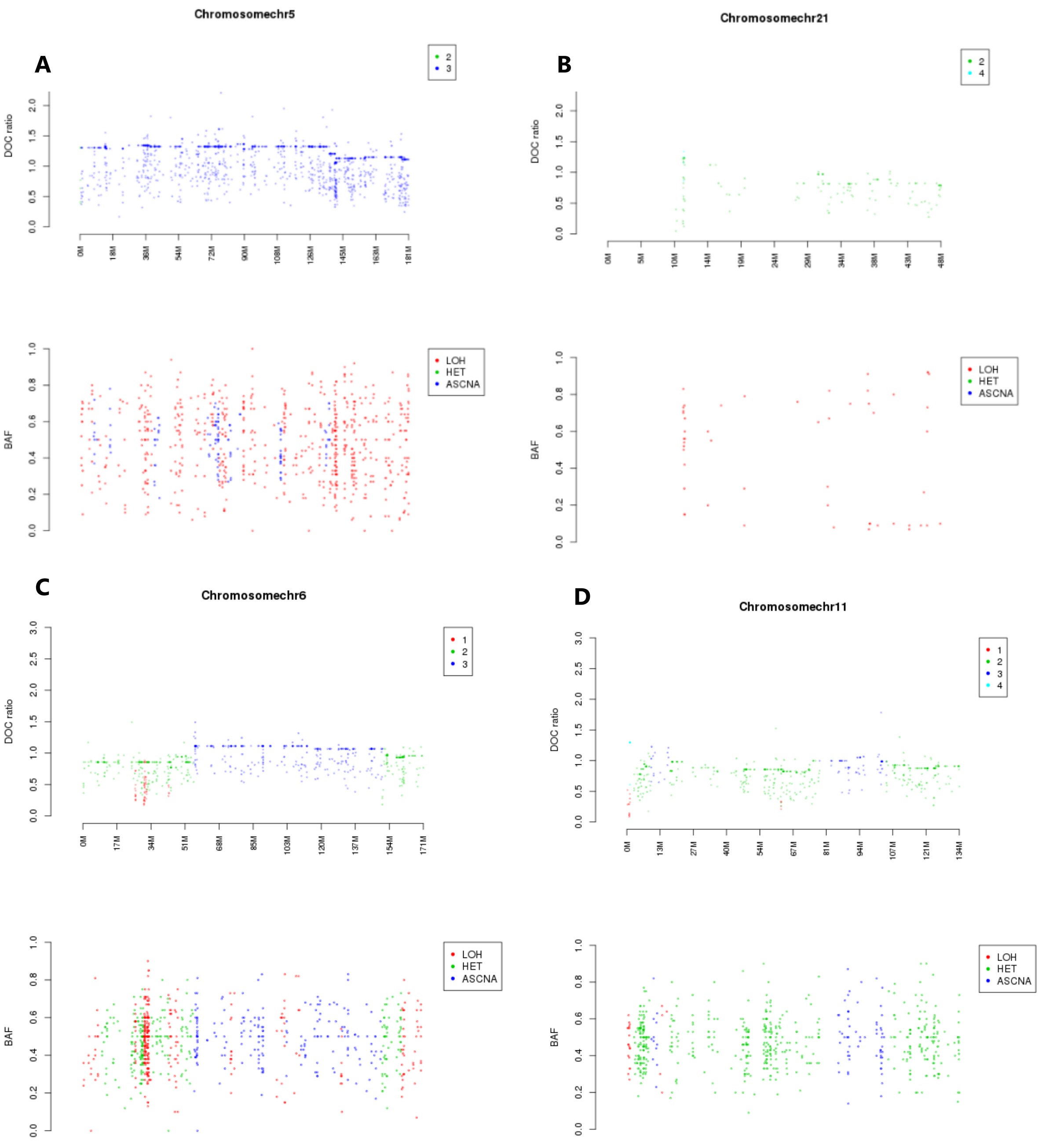

Supplementary Figure S1: ADTEx copy number analysis of Biopsy 1 from low coverage WES
Copy number profile of low-coverage WES of Biopsy 1 revealing **A,** likely amplification of Chr 5 and **B,** loss of Chr 21. Top panels display DOC ratio, colored by predicted copy number state. Bottom panels display BAF, colored by predicted copy number alteration. The separation of BAF towards 0.3 and 0.6 in Chr 5 indicates chromosomal gain, whereas the separation of BAF towards 0 and 1 in Chr 21 indicates LoH. There was insufficient evidence conclusively identify the presence or absence of the loss of **C,** Chr 6 and **D,** chromothripsis of one copy of Chr 11 based on ADTEx analysis of the low-coverage WES of Biopsy 1, however it is likely that they were absent. Copy number analysis was plagued with high levels of noise from low coverage and large differences in coverage between the Biopsy 1 and normal sample. The sole Chr 11 breakpoint covered by the exome sequencing did not provide read support for chromothripsis. The apparent amplifications of regions of each chromosome do not match up to any known alterations in high-quality DNA samples from tumours collected at autopsy and are likely attributable to noise in the low coverage WES.
