# Supplementary material for: Complex Patterns of Genomic Heterogeneity Identified in 42 Tumor Samples and ctDNA of a Pulmonary Atypical Carcinoid Patient
Source: Cancer Res Commun. 2023 Jan 10;3(1):31–42. doi: 10.1158/2767-9764.CRC-22-0101 (PMC10035512; doi:10.1158/2767-9764.CRC-22-0101)
Supplement: Supplementary Figure S2 — Breakpoints in 10x Loupe software linked-reads view. Each bar represents a sequencing read, and those joined with a horizontal line share the same barcode. Reads are grouped by haplotype (green and purple), with unphased reads in grey. Vertical orange lines indicate putative breakpoints defined by the software. Given that the maximum region of the chromosome phased was significantly shorter than the length of Chr 11 (as indicated in the ‘phase block view’ plot), some breakpoints are assigned to different haplotypes, therefore haplotypes should not be compared between regions (the breakpoint appears in purple in some regions and in green in others). The copy number patterns are consistent with all breakpoints occurring on the same copy of the chromosome. [file crc-22-0101-s03.pdf]

Chr11:23,390,024

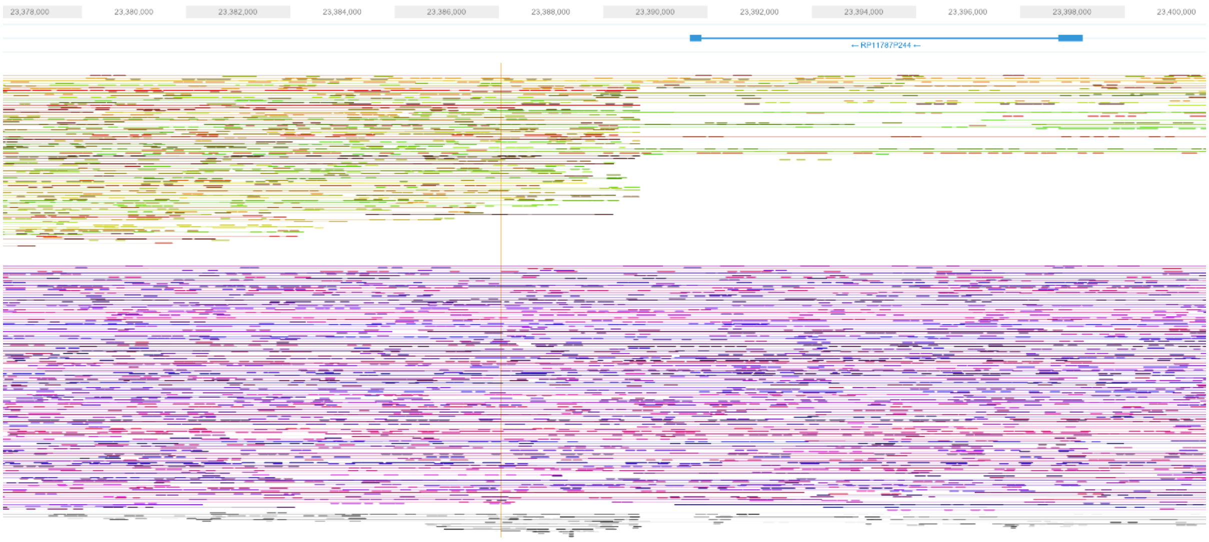

Chr11:50,093,818

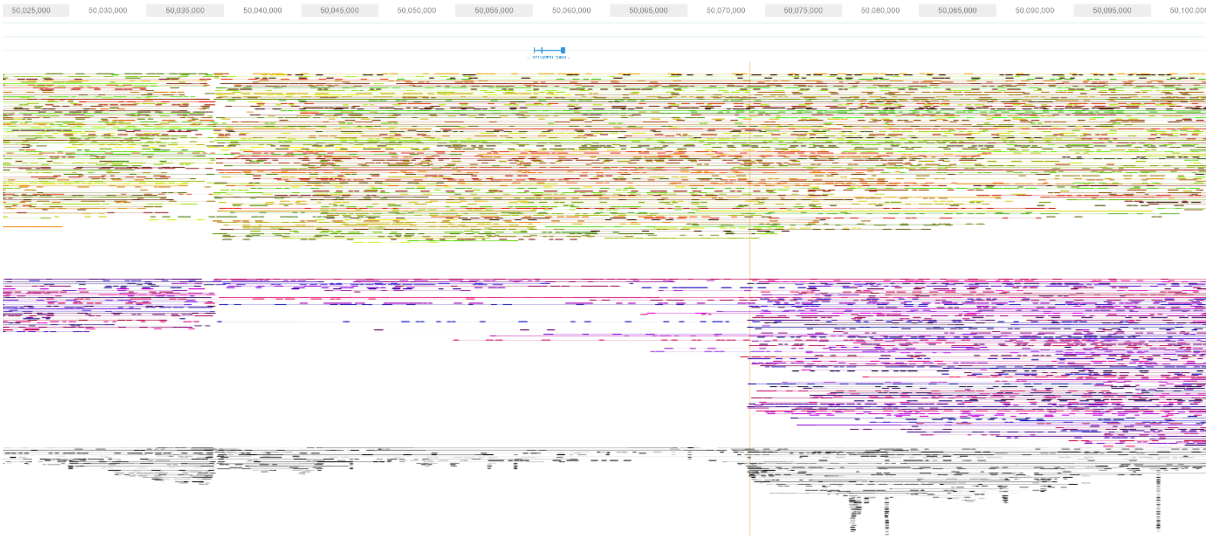

Chr11:56,040,991

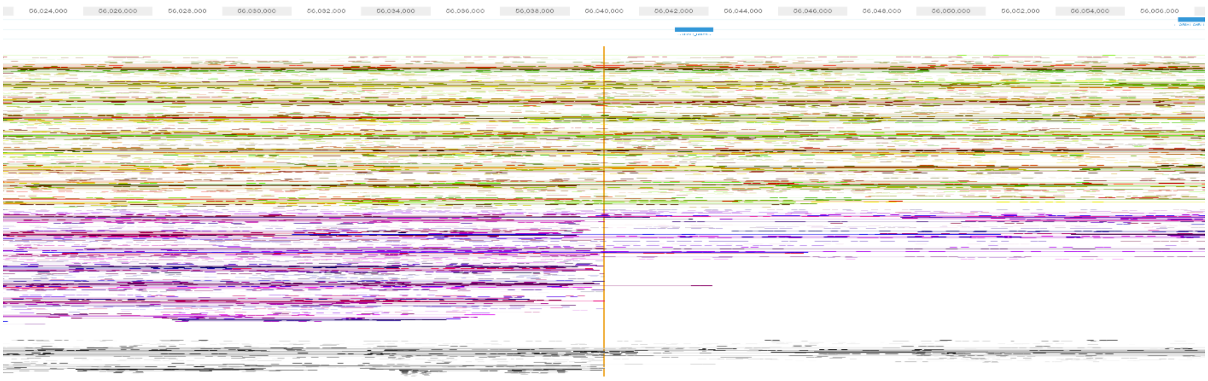

Chr11: 91,895,856

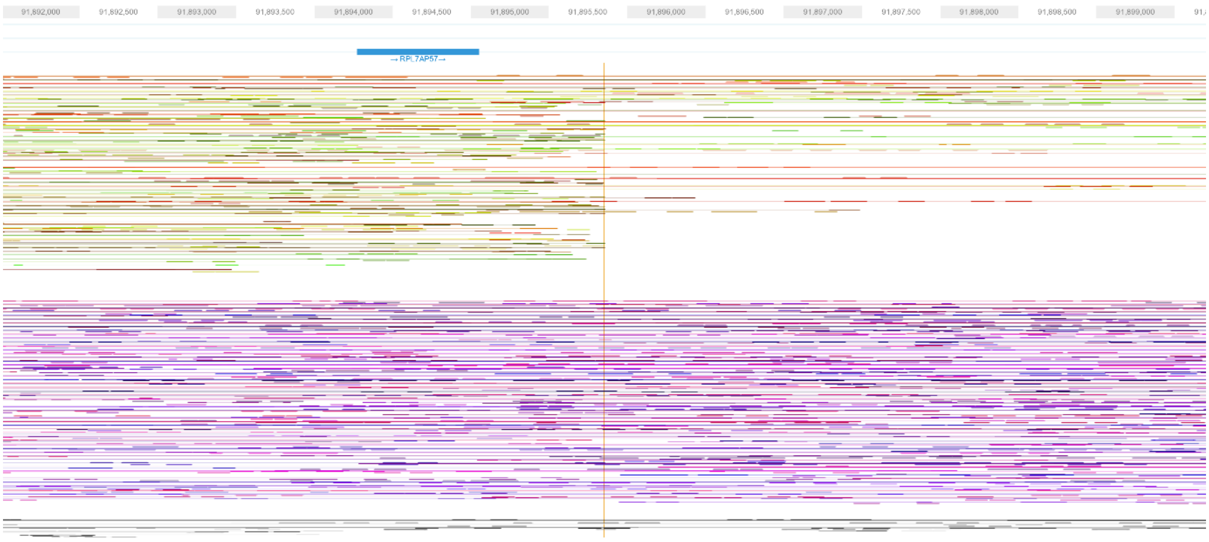

Chr11:85,162,609

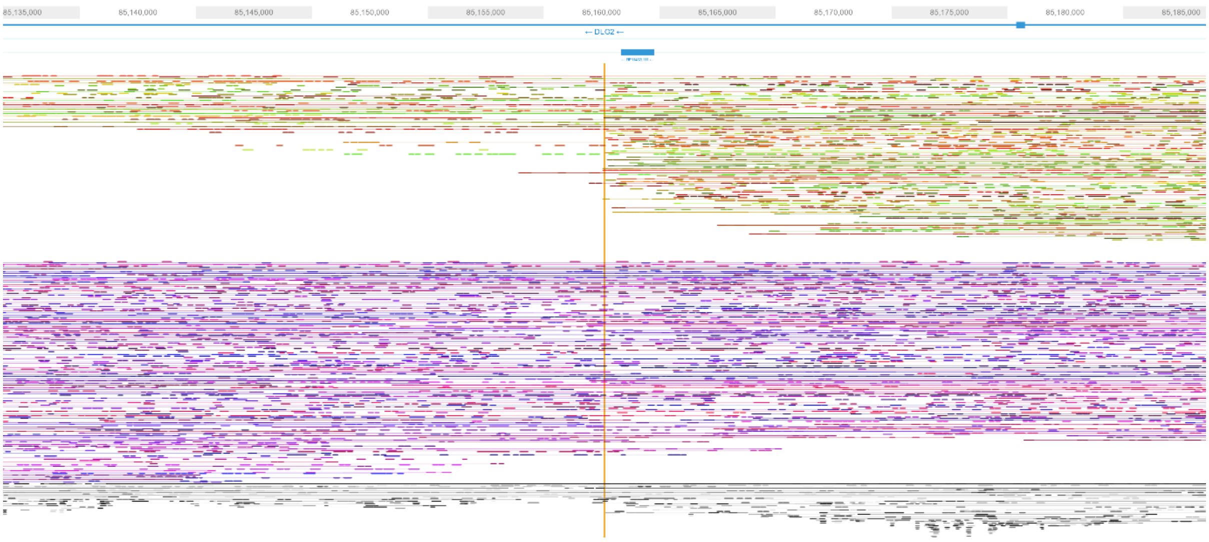

Chr11:67,833,983

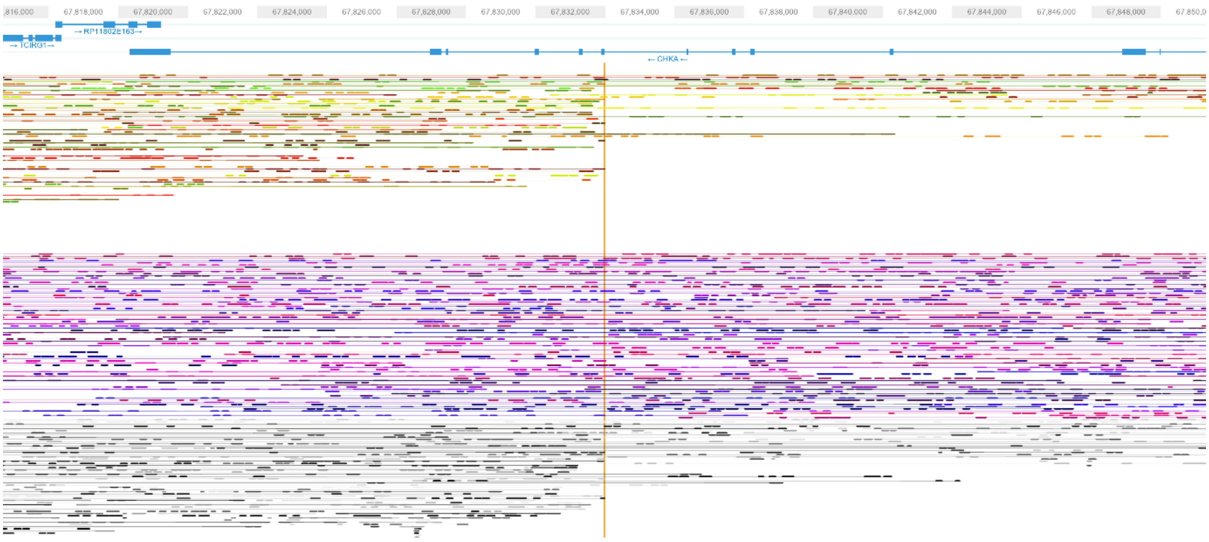

Chr11:63,355,723

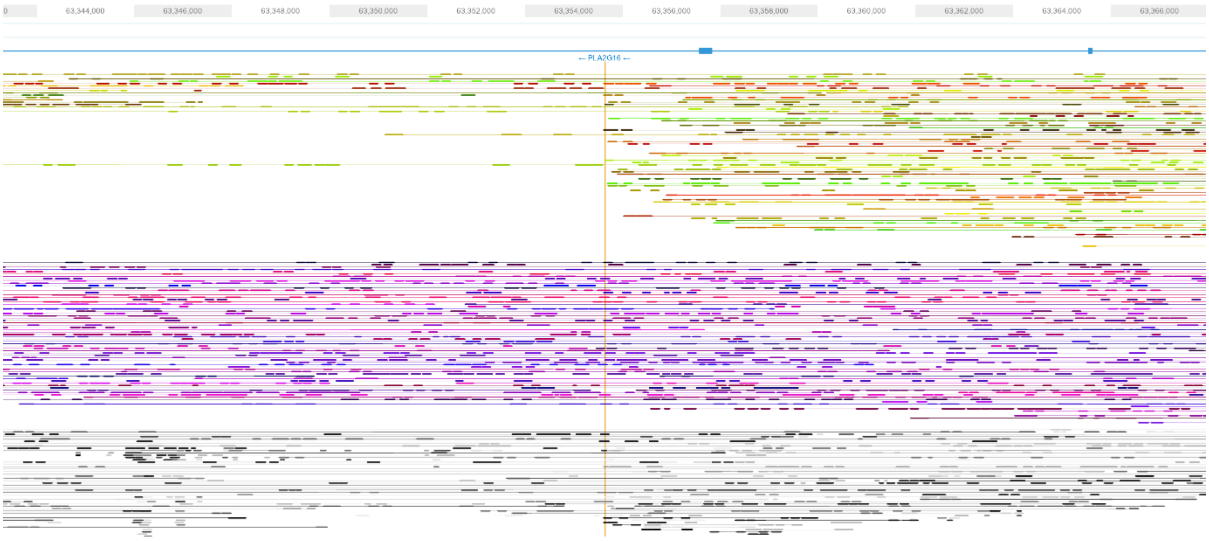

Chr11:28,575,572

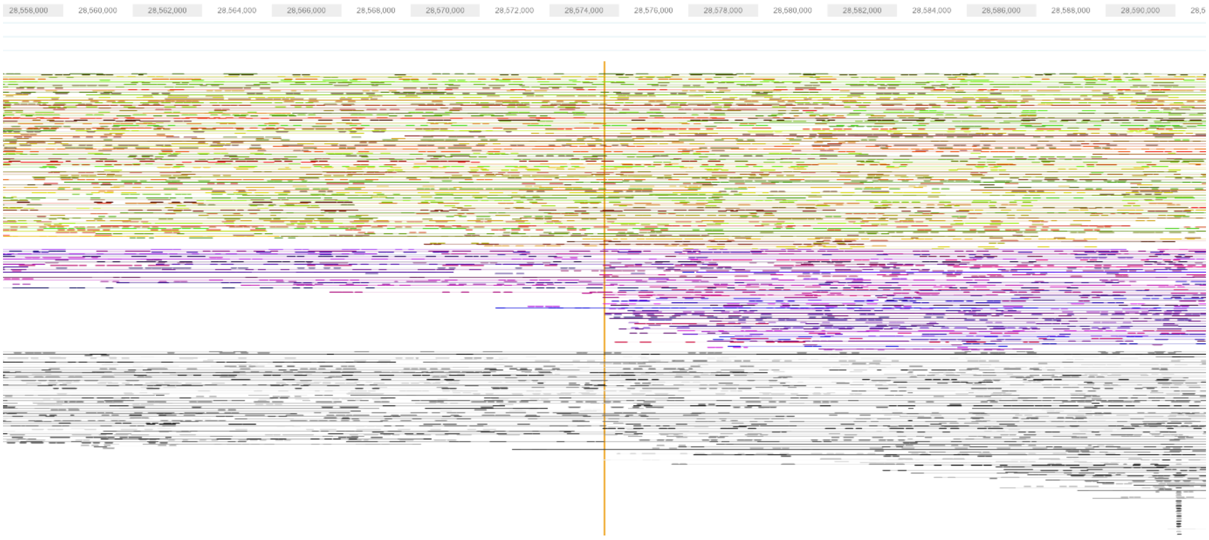

Chr11:36,677,007

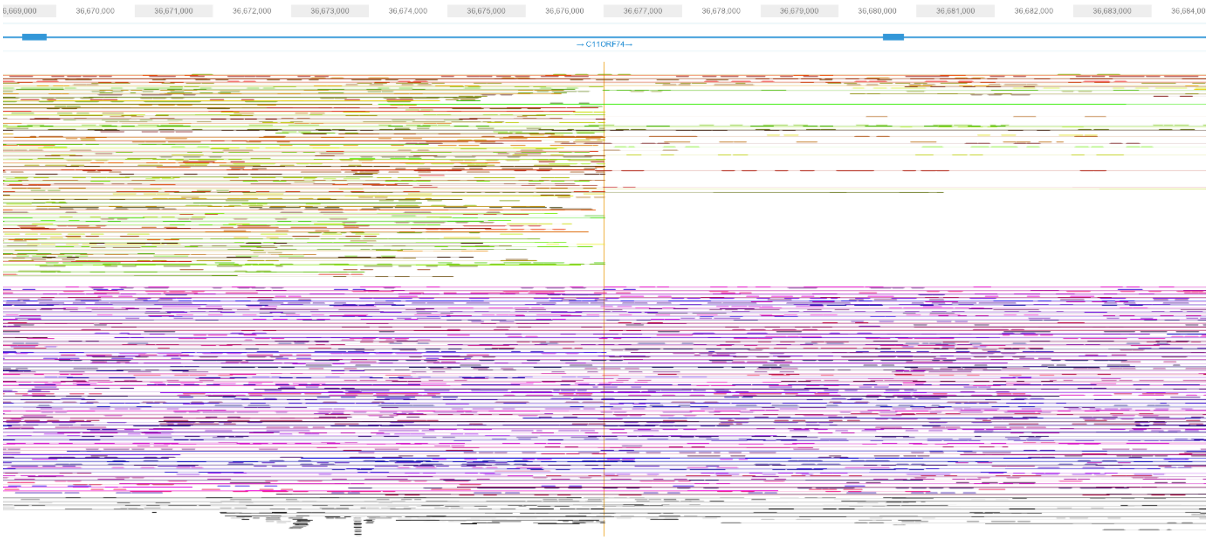

Chr11:105,797,381

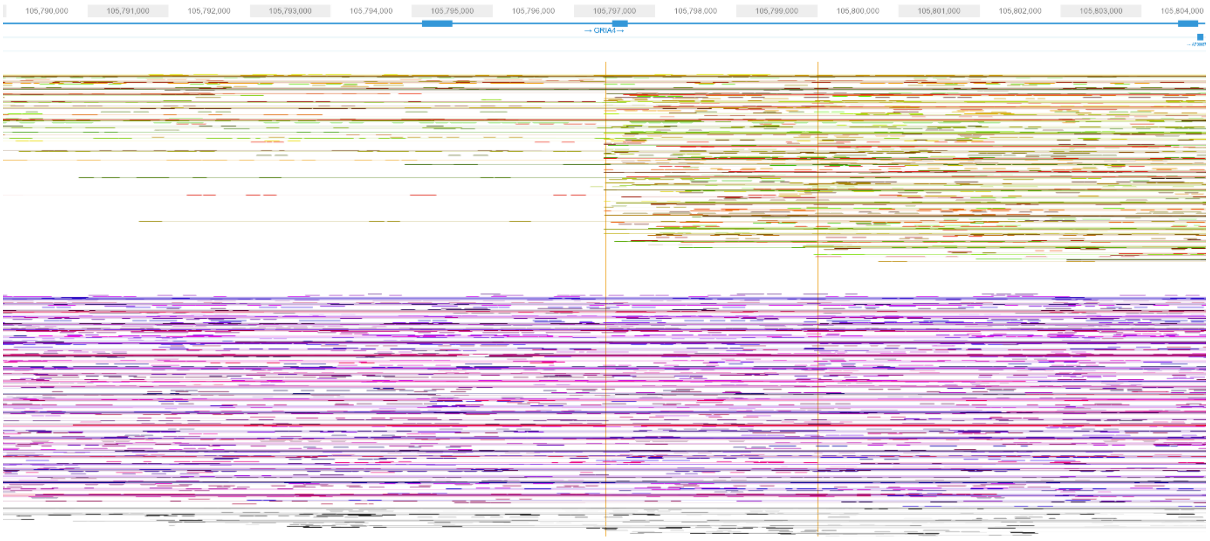

Chr11:109,294,342

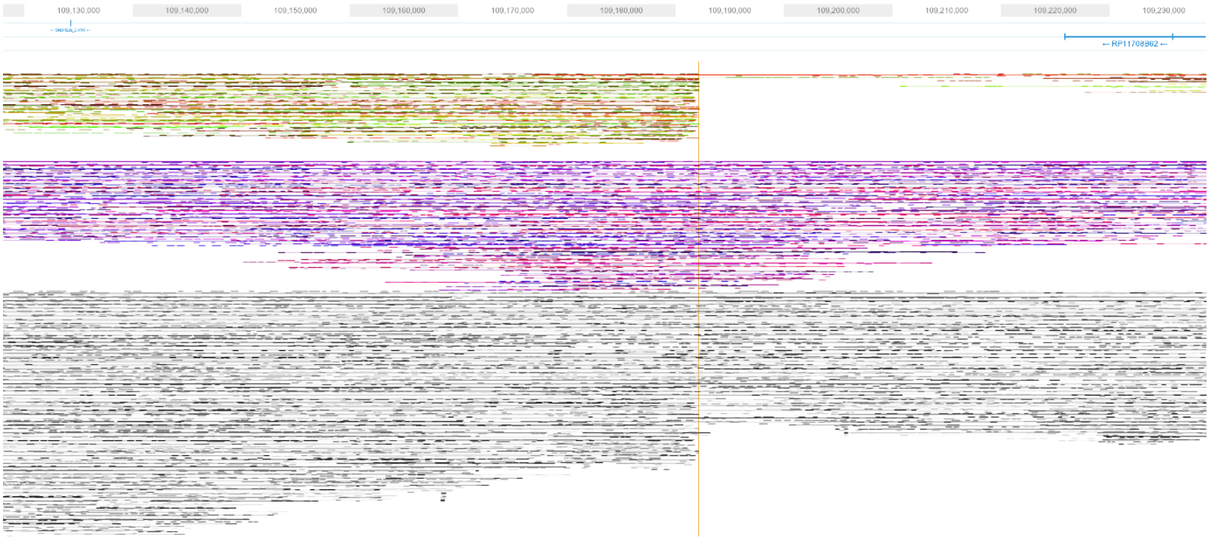

Phase block view

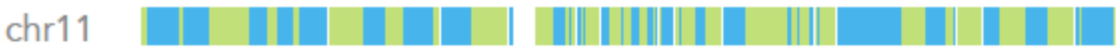

Supplementary Figure S2: Breakpoints in 10x Loupe software linked-reads view.
Each bar represents a sequencing read, and those joined with a horizontal line share the same barcode. Reads are grouped by haplotype (green and purple), with unphased reads in grey. Vertical orange lines indicate putative breakpoints defined by the software. Given that the maximum region of the chromosome phased was significantly shorter than the length of Chr 11 (as indicated in the 'phase block view' plot), some breakpoints are assigned to different haplotypes, therefore haplotypes should not be compared between regions (the breakpoint appears in purple in some regions and in green in others). The copy number patterns are consistent with all breakpoints occurring on the same copy of the chromosome.
