# Supplementary material for: Complex Patterns of Genomic Heterogeneity Identified in 42 Tumor Samples and ctDNA of a Pulmonary Atypical Carcinoid Patient
Source: Cancer Res Commun. 2023 Jan 10;3(1):31–42. doi: 10.1158/2767-9764.CRC-22-0101 (PMC10035512; doi:10.1158/2767-9764.CRC-22-0101)
Supplement: Supplementary Figure S3 — Mutational signatures across all samples collected at autopsy A, Overall mutational burden does not reveal significant mutational signatures (MuSiCa). The signature contribution is indicated by the degree of shading (key to right). The signature with the highest contribution was Signature 3, however it was not statistically significant in most samples according to tool Signal. B, The scarHRD method for quantifying homologous recombination deficiency did not identify any tumors to be HR-deficient; with all having an HRD-sum score < 20 (known HR-deficient tumors usually have HRD-sums of > 40)28. [file crc-22-0101-s04.pdf]

A

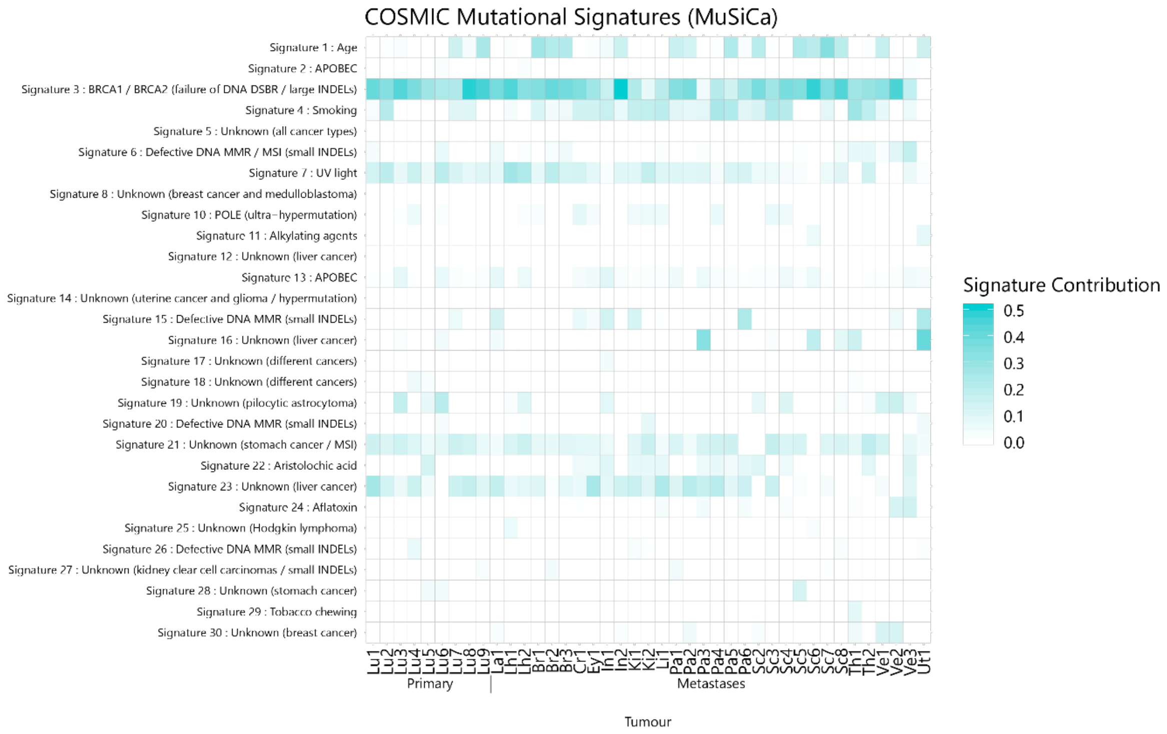

B

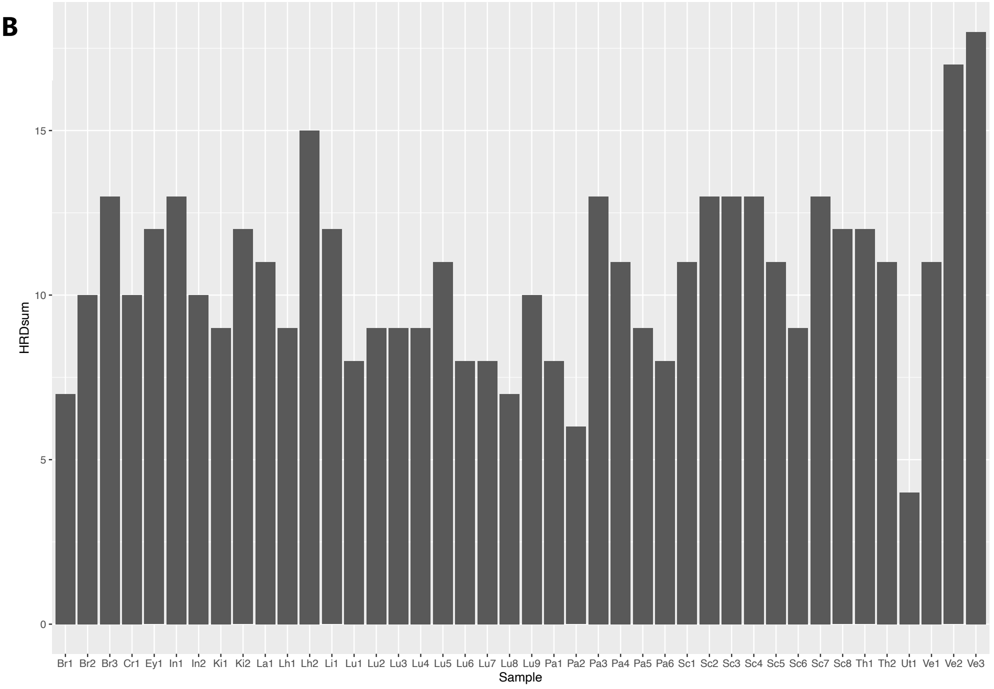

Supplementary Figure S3: Mutational signatures across all samples collected at autopsy

A, Overall mutational burden does not reveal significant mutational signatures (MuSiCa). The signature contribution is indicated by the degree of shading (key to right). The signature with the highest contribution was Signature 3, however it was not statistically significant in most samples according to tool Signal. **B,** The scarHRD method for quantifying homologous recombination deficiency did not identify any tumors to be HR-deficient; with all having an HRD-sum score < 20 (known HR-deficient tumors usually have HRD-sums of > 40)[28].
