# Supplementary material for: Complex Patterns of Genomic Heterogeneity Identified in 42 Tumor Samples and ctDNA of a Pulmonary Atypical Carcinoid Patient
Source: Cancer Res Commun. 2023 Jan 10;3(1):31–42. doi: 10.1158/2767-9764.CRC-22-0101 (PMC10035512; doi:10.1158/2767-9764.CRC-22-0101)
Supplement: Supplementary Figure S4 — Absence of ARID1A variant in targeted panel sequencing of Biopsy 1 despite adequate depth Top panel shows Biopsy 1 targeted panel sequencing. Bottom panel shows representative tumor sampled at autopsy (Sc6) with heterozygous deletion in ARID1A, clearly absent from Biopsy 1 sequencing despite adequate depth (7240 unique molecules). The probability of not sampling the ARID1A variant in Biopsy 1 due to chance alone was calculated assuming tumor cellularity of 80% and the heterozygous ARID1A variant being present in 50% of tumor reads. 7240 unique molecules covered this genomic position. The binomial distribution in R was used to calculate the probability of not sampling this variant: dbinom(7240, size=7240, prob=0.6). [file crc-22-0101-s05.pdf]

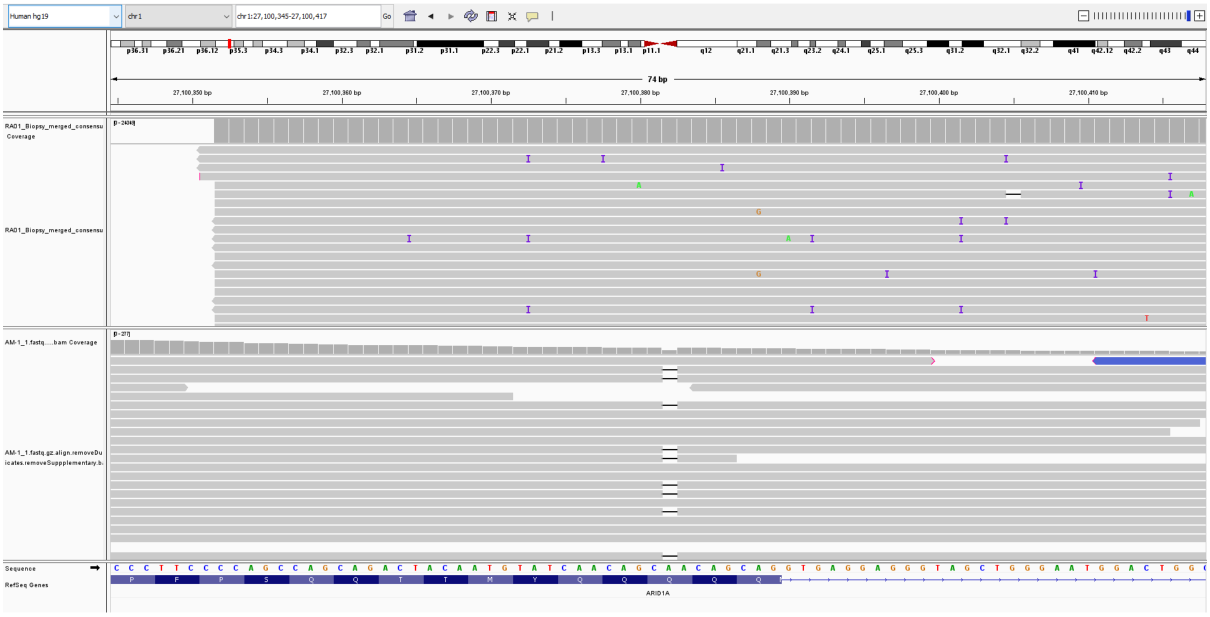

Supplementary Figure S4: Absence of *ARID1A* variant in targeted panel sequencing of Biopsy 1 despite adequate depth

Top panel shows Biopsy 1 targeted panel sequencing. Bottom panel shows representative tumor sampled at autopsy (Sc6) with heterozygous deletion in *ARID1A*, clearly absent from Biopsy 1 sequencing despite adequate depth (7240 unique molecules). The probability of not sampling the *ARID1A* variant in Biopsy 1 due to chance alone was calculated assuming tumor cellularity of 80% and the heterozygous *ARID1A* variant being present in 50% of tumor reads. 7240 unique molecules covered this genomic position. The binomial distribution in R was used to calculate the probability of not sampling this variant: dbinom(7240,size=7240,prob=0.6).
