# Supplementary material for: Complex Patterns of Genomic Heterogeneity Identified in 42 Tumor Samples and ctDNA of a Pulmonary Atypical Carcinoid Patient
Source: Cancer Res Commun. 2023 Jan 10;3(1):31–42. doi: 10.1158/2767-9764.CRC-22-0101 (PMC10035512; doi:10.1158/2767-9764.CRC-22-0101)
Supplement: Supplementary Figure S5 — Inference of putative sequences of genomic changes consistent with the data using the LICHeE and REVOLVER methods LICHeE and Revolver were used to further speculate the order of genomic changes in this patient's dataset. ClonEvol was also trialed, using PyClone putative clonal clusters as input, however the results did not converge. A, LICHeE was run according to the tool creator's instructions, using VAFs as input, to generate clonal relationships of tumor samples. Numbers inside colored circles represent the number of mutations defining a clone, and the squares represent the clonal structures of individual samples. Shaded regions indicate the proportion of cells belonging to that clone, where the white regions represent normal cells. Four dominant tumor groups were apparent. Where two or more colors are present, LICHeE has predicted the sample to contain a mixture of clones (most apparent in Pa1 and Lu1 but also present in In2, Pa6 and others). This feature is also visible in the VAF plot (Figure 5a) but not represented on the DNA phylogram (Figure 5b). Technical reasons prevented the inclusion of some samples, e.g. Lu8 and Pa5. B, Clonal relationships between tumor samples as predicted by REVOLVER, using PyClone putative clonal clusters as input (from VAF and chromosomal copy number), and default tool parameters. Clones are labeled according to one variant defining that clone (e.g., MASTL), and the circle size is proportional to the number of variants defining the clone. Number refers to clone number. REVOLVER highlighted the same pattern of progression seen in other analyses: from normal cells, common variants accumulate (labeled as MASTL in Figure 5b), and a further set of variants shared by most tumors was identified (labeled GPAM) before a split into two dominant tumor groups, each characterized by their own set of variants (labeled SLIT1 and PAQR6 respectively). REVOLVER highlights the progression of variant accumulation across all s [file crc-22-0101-s06.pdf]

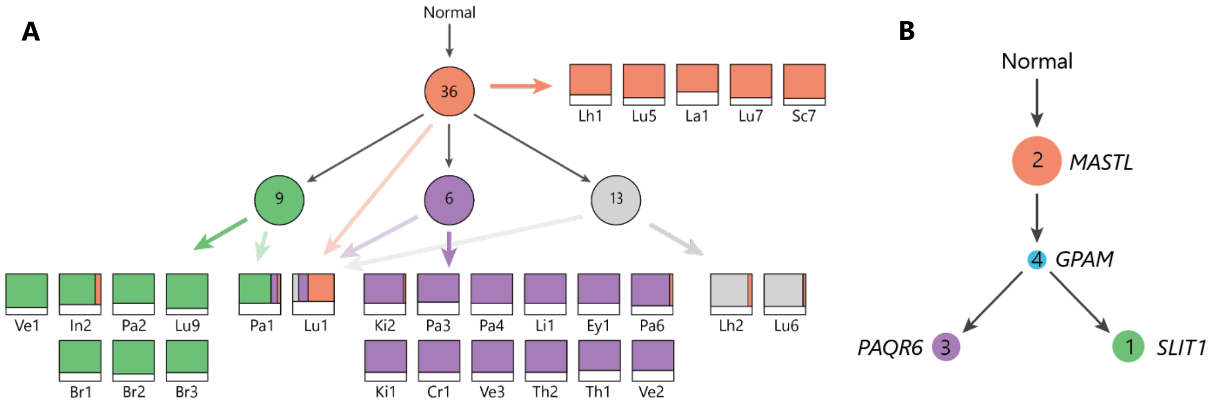

Supplementary Figure S5: Inference of putative sequences of genomic changes consistent with the data using the LICHeE and REVOLVER methods

LICHeE and Revolver were used to further speculate the order of genomic changes in this patient's dataset. ClonEvol was also trialed, using PyClone putative clonal clusters as input, however the results did not converge. **A,** LICHeE was run according to the tool creator's instructions, using VAFs as input, to generate clonal relationships of tumor samples. Numbers inside colored circles represent the number of mutations defining a clone, and the squares represent the clonal structures of individual samples. Shaded regions indicate the proportion of cells belonging to that clone, where the white regions represent normal cells. Four dominant tumor groups were apparent. Where two or more colors are present, LICHeE has predicted the sample to contain a mixture of clones (most apparent in Pa1 and Lu1 but also present in In2, Pa6 and others). This feature is also visible in the VAF plot (Figure 5a) but not represented on the DNA phylogram (Figure 5b). Technical reasons prevented the inclusion of some samples, e.g. Lu8 and Pa5. **B,** Clonal relationships between tumor samples as predicted by REVOLVER, using PyClone putative clonal clusters as input (from VAF and chromosomal copy number), and default tool parameters. Clones are labeled according to one variant defining that clone (e.g., *MASTL*), and the circle size is proportional to the number of variants defining the clone. Number refers to clone number. REVOLVER highlighted the same pattern of progression seen in other analyses: from normal cells, common variants accumulate (labeled as *MASTL* in Figure 5b), and a further set of variants shared by most tumors was identified (labeled *GPAM*) before a split into two dominant tumor groups, each characterized by their own set of variants (labeled *SLIT1* and *PAQR6* respectively). REVOLVER highlights the progression of variant accumulation across all samples, rather than indicating individual samples. All coloring consistent with Figure 5. Overall, LICHeE and REVOLVER explore the clonal and subclonal structure of individual tumor samples and suggest the same sequence of genomic progression evident in the DNA phylogram (Figure 5b). None of these cancer-specific methods attempts to time the evolutionary divergence events.
