# Supplementary material for: Complex Patterns of Genomic Heterogeneity Identified in 42 Tumor Samples and ctDNA of a Pulmonary Atypical Carcinoid Patient
Source: Cancer Res Commun. 2023 Jan 10;3(1):31–42. doi: 10.1158/2767-9764.CRC-22-0101 (PMC10035512; doi:10.1158/2767-9764.CRC-22-0101)
Supplement: Supplementary Figure S6 — ctDNA detection of private variants While only four private variants were reported by the sequencing platform’s IonReporter software (indicated in orange, key top right), there were sequencing reads to support other private variants. Private variants unique to samples La1 (3 variants) and Pa6 (1 variant) were supported by 2 to 3 variant molecules, and there was a single variant molecule detected to support private variant detection in the following samples: Br2, Cr1, In2, Ki2, La1, Lh1, Lh2, Lu6, Lu7, Lu9, Pa2, Pa3, Pa6, Sc7, Th1, Th2, Ut1, Ve2, Ve3. [file crc-22-0101-s07.pdf]

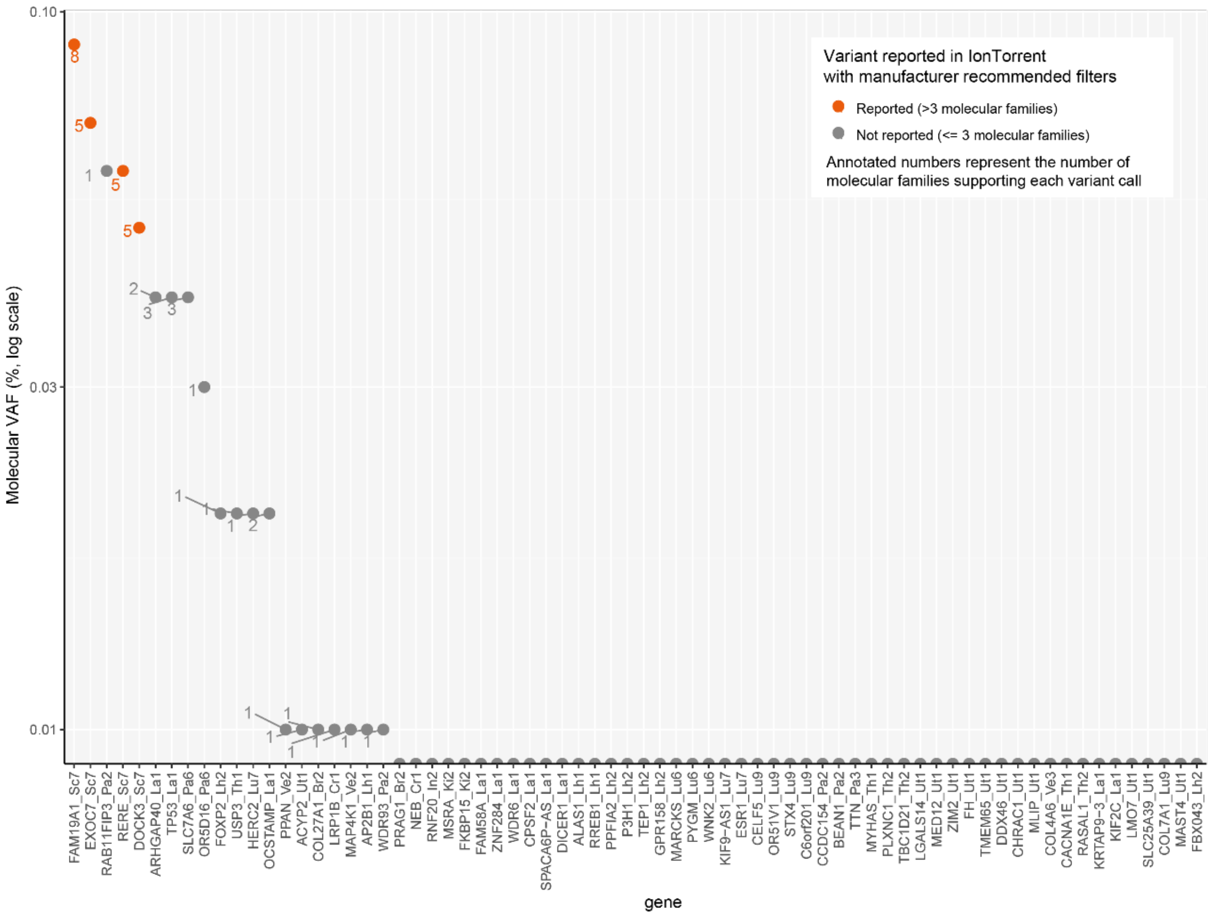

Supplementary Figure S6: ctDNA detection of private variants

While only four private variants were reported by the sequencing platform's IonReporter software (indicated in orange, key top right), there were sequencing reads to support other private variants. Private variants unique to samples La1 (3 variants) and Pa6 (1 variant) were supported by 2 to 3 variant molecules, and there was a single variant molecule detected to support private variant detection in the following samples: Br2, Cr1, In2, Ki2, La1, Lh1, Lh2, Lu6, Lu7, Lu9, Pa2, Pa3, Pa6, Sc7, Th1, Th2, Ut1, Ve2, Ve3.
